# Insight into the Binding and Hydrolytic Preferences of hNudt16 Based on Nucleotide Diphosphate Substrates

**DOI:** 10.3390/ijms222010929

**Published:** 2021-10-10

**Authors:** Magdalena Chrabąszczewska, Maria Winiewska-Szajewska, Natalia Ostrowska, Elżbieta Bojarska, Janusz Stępiński, Łukasz Mancewicz, Maciej Łukaszewicz, Joanna Trylska, Michał Taube, Maciej Kozak, Edward Darżynkiewicz, Renata Grzela

**Affiliations:** 1 Division of Biophysics, Institute of Experimental Physics, Faculty of Physics, University of Warsaw, Pasteura 5, 02-093 Warsaw, Poland; Magdalena.Chrabaszczewska@fuw.edu.pl (M.C.); lu.mancewicz@gmail.com (Ł.M.); Maciej.Lukaszewicz@fuw.edu.pl (M.Ł.); edward.darzynkiewicz@cent.uw.edu.pl (E.D.); 2 Institute of Biochemistry and Biophysics, Polish Academy of Sciences, Pawinskiego 5a, 02-106 Warsaw, Poland; mwin@ibb.waw.pl; 3 Centre of New Technologies, University of Warsaw, Banacha 2c, 02-097 Warsaw, Poland; n.ostrowska@cent.uw.edu.pl (N.O.); e.bojarska@cent.uw.edu.pl (E.B.); j.stepinski@cent.uw.edu.pl (J.S.); joanna@cent.uw.edu.pl (J.T.); 4 Department of Macromolecular Physics, Faculty of Physics, Adam Mickiewicz University, Uniwersytetu Poznanskiego 2, 61-614 Poznan, Poland; mtaube@amu.edu.pl (M.T.); mkozak@amu.edu.pl (M.K.); 5 National Synchrotron Radiation Centre SOLARIS, Jagiellonian University, Czerwone Maki 98, 30-392 Krakow, Poland

**Keywords:** hNudt16, nudix family, dinucleoside diphosphates, GppG, IDP, MST, DSF, SAXS

## Abstract

Nudt16 is a member of the NUDIX family of hydrolases that show specificity towards substrates consisting of a nucleoside diphosphate linked to another moiety X. Several substrates for hNudt16 and various possible biological functions have been reported. However, some of these reports contradict each other and studies comparing the substrate specificity of the hNudt16 protein are limited. Therefore, we quantitatively compared the affinity of hNudt16 towards a set of previously published substrates, as well as identified novel potential substrates. Here, we show that hNudt16 has the highest affinity towards IDP and GppG, with K_d_ below 100 nM. Other tested ligands exhibited a weaker affinity of several orders of magnitude. Among the investigated compounds, only IDP, GppG, m^7^GppG, AppA, dpCoA, and NADH were hydrolyzed by hNudt16 with a strong substrate preference for inosine or guanosine containing compounds. A new identified substrate for hNudt16, GppG, which binds the enzyme with an affinity comparable to that of IDP, suggests another potential regulatory role of this protein. Molecular docking of hNudt16-ligand binding inside the hNudt16 pocket revealed two binding modes for representative substrates. Nucleobase stabilization by Π stacking interactions with His24 has been associated with strong binding of hNudt16 substrates.

## 1. Introduction

The human Nudt16 protein (hNudt16) is a member of the NUDIX family of enzymes involved in cellular metabolism, homeostasis, and mRNA processing. As NUDIX refers to a nucleoside diphosphate linked to another moiety X, the enzymes of this family catalyze the hydrolysis of the phosphodiester bond in a broad spectrum of substrates (including nucleoside triphosphates, coenzymes, nucleotide sugars, dinucleoside polyphosphates) [1,2]. The Nudix family of enzymes have a highly conserved 23-residue sequence motif called the Nudix box (GX5EX7REUXEEXGU), where X may be any residue and U represents hydrophobic residues [1,3]. The structural motif of the Nudix box (loop–α helix–loop) acts as a substrate-binding and catalytic site. It is also involved in the binding of metal ions (Mg^2+^, Mn^2+^, or Co^2+^), which are essential for substrate hydrolysis [2,4,5,6,7]. hNudt16 orthologs have been identified in numerous eukaryotic species, including vertebrates (e.g., frog, rat, zebra fish, and lamprey) and invertebrates (e.g., earthworms) [8,9,10], indicating an evolutionarily conserved biological role [8].

hNudt16 has been reported to hydrolyze the canonical 5′-RNA cap structure on a subset of cytoplasmic mRNAs and small nucleolar RNAs, releasing m^7^GDP or $m_{3}^{2,2,7}$GDP and 5′-monophosphate RNA [5,8,9,10]. As hNudt16 activity overlaps with the hydrolytic activity of Dcp2 in mammalian cells, it has been proposed that hNudt16 may act as a second decapping enzyme involved in the 5′ → 3′ degradation of RNAs [11]. However, studies in our laboratory have shown that hNudt16 prefers unmethylated cap RNAs as substrates rather than standard m^7^G bearing transcripts [12]. Additionally it should be emphasized that hydrolysis of both cap structure analogues (m^7^GpppG/A or GpppG/A) and short RNAs capped with such analogues required high hNudt16 concentrations and long reaction times. Other studies have reported that hNudt16 hydrolyzes non-canonical metabolites (NAD, dpCoA, FAD) present at the 5′-end of some cellular RNAs [13,14]. hNudt16 has also been linked to the maintenance of chromosome stability and cell growth, with the ability to eliminate cell-damaging dIDP/IDP and dITP/ITP [15,16,17]. Recent publications have reported a possible role for hNudt16 in protein ADP-ribosylation processing, as well as in the hydrolysis of mono- and poly-ADP-ribosylation on RNA or DNA substrates [14,18,19,20,21,22]. The activity of hNudt16 has been shown to be essential for 53BP1 de-ADP-ribosylation, and its deficiency has been found to affect 53BP1 stability and function [23]. Some of the above reports contradict the data presented in [24]. The authors presented a substrate redundancy map for different members of the human NUDIX hydrolase family. Among the substrates they studied were: ADP-ribose, CoA, ApppA, GDP, GTP, ITP, m^7^GpppG, NAD^+^. Surprisingly, none of the compounds listed were classified as substrate for hNudt16 but instead were identified as specific for other members of the NUDIX hydrolase family, specifically ADP-ribose for Nudt5/Nudt9/Nudt12/Nudt14, ApppA for Nudt12/Nudt14, GDP for Nudt18, GTP for Nudt15, ITP for Nudt15, NAD^+^ for Nudt12. No specific enzymatic activity against CoA and m^7^GpppG was detected among the proteins studied. Moreover, the authors did not propose any clear substrate for Nudt16.

Biochemical and biophysical studies on different classes of substrates for hNudt16 (purine containing nucleotides, dinucleotides bearing triphosphate bridge, and short ribo-oligonucleotides) have identified the main determinants for substrate recognition [10,12,16,17,20]. Structural data for hNudt16 with ligands are available for IMP (PDB:2XSQ), ADP-ribose (PDB:5W6X), diADPR (PDB:6B09), and FAD (PDB:6X7U). All these ligands are located inside the main binding pocket of hNudt16 in the vicinity of the metal ions. Several key residues that are crucial for hNudt16 activity are also located there [5,10,25]. Mutations R75L or E79Q have been found to reduce the enzymatic efficiency of the protein, and single mutations E76Q and E80Q, or the cluster double mutation E79K and E80K (EE-KK), can cause a complete loss of hydrolytic activity [10,13]. However, the effects of these mutations on ligand-binding properties have yet to be studied.

Given the inconsistent data on hNudt16 activity, we decided to investigate the substrate specificity of this protein. Our aim was to compare the specificity of hNudt16 towards different published substrates under the same experimental conditions to obtain quantitative data. For some published substrates, quantitative data are lacking, making very difficult to assess the significance of enzyme activity against this compound. It is necessary to systematize the data with a quantitative assessment of hNudt16 activity in order to further determine the biological role of this enzyme. We also decided to test hNudt16 activity against several new compounds that we found interesting.

Here, we evaluated hNudt16 hydrolysis towards dinucleotide-containing diphosphates. Dinucleoside polyphosphate (XpnX; X = adenosine, guanosine, inosine or uridine, *n* = 2–7) have been shown to acts as extracellular mediators controlling numerous physiological functions, e.g., vascular tone or cell proliferation [26]. Two representatives of this group, GppG and AppA, were identified in human platelets and adrenal glands [26,27]. Dinucleoside polyphosphate, mononucleosides, and mononucleoside polyphosphates together with enzymes and their corresponding receptors form a purinergic signaling system [28]. Guanidine-based purines (GTP, GDP, GMP, GUO, GUA) as a part of this system, have been shown to modulate intracellular processes such as growth, differentiation and survival [29]. It is therefore important to study the activity of hNudt16 against dinucleoside diphosphate, as this enzyme may be involved in controlling the levels of these compound, which affect important cellular processes. Our data showed a very fast catalytic process rate for the GppG compound. To have a more complete insight into substrate selection we decided to study not only the hydrolysis process but also the binding affinity of hNudt16 to broader list of selected substrates. Therefore, we investigated and compared the binding affinity to di- and triphosphate nucleosides, dinucleotides and metabolites containing different bases (adenine, hypoxanthine, and guanine) for a set of ligands previously reported as hNudt16 substrates: m^7^GpppG [13,30], NADH [30], NAD [13,30] and dpCoA [13], GDP [16], IDP [15,16,17], free ADPr [19,20,21,22,23], GpppG [13], and CDP [16], novel unpublished compounds: GppG, m^7^GppG, m^7^GDP, and AppA, as well as control monophosphate compounds IMP (inhibitor with known binding parameters [17]) and GMP. We focused on free compounds because in our previous work we did not observe significant difference in hydrolysis when compounds (m^7^GpppG or GpppG) were bound to the RNA chain [12]. To study the binding affinity, we used the E76Q hNudt16 mutant, which shows impaired hydrolytic activity. To gain insights into the position of the different ligands with respect to the hNudt16 structure, we performed molecular docking of selected ligands to the hNudt16 crystal structure.

## 2. Results

### 2.1. Hydrolytic Activity of hNudt16 on Dinucleotides Containing Diphosphate Bridge

**Figure 1:**
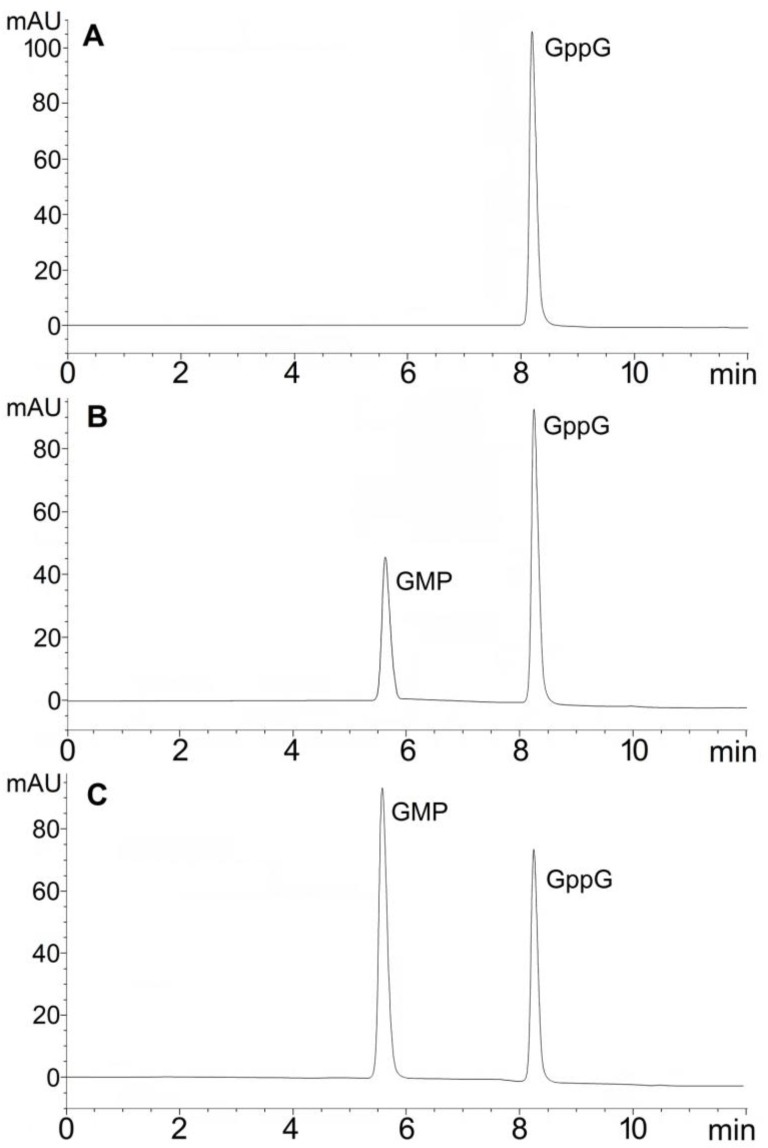
HPLC chromatograms for the hydrolysis of GppG catalyzed by hNudt16. (**A**) GppG in buffer (without hNudt16), (**B**) after 2 min reaction with hNudt16, (**C**) after 8 min reaction with hNudt16. Substrate concentration was 20 µM, enzyme concentration 0.002 µM. Chromatogram (**A**) was obtained after five-minute substrate incubation at 95 °C to show that heat inactivation of the enzyme in the assay buffer does not lead nonenzymatic degradation of GppG.

We have started our research by investigating the hydrolysis of diphosphate compounds. We were interested in whether GppG and AppA and their methylated counterparts are processed by hNudt16. We also performed measurements for previously published hNudt16 substrates, namely IDP, m^7^GDP, ADPr, dpCoA, NAD, and NADH. The hydrolysis reactions of all compounds were studied under the same experimental conditions (substrate concentration, buffer composition, and temperature). The products were analyzed and quantified using HPLC. Representative chromatograms for GppG are presented in Figure 1, and for m^7^GppG and AppA are shown in Figure S1. All investigated dinucleotides were hydrolyzed by hNudt16, while ADPr and m^7^GDP proved resistant to hydrolytic cleavage under the conditions tested (Table 1). The data presented in Table 1 indicate that unmethylated dinucleotide GppG was very efficiently hydrolyzed by hNudt16. The hydrolysis rate for GppG was much higher than that of its m^7^GppG counterpart. Dinucleotides containing adenine, AppA, dpCoA, NAD, and NADH were much less susceptible to enzymatic cleavage by this enzyme. These results are consistent with our previous report showing that dinucleotides containing adenine instead of guanine or methylated at the N7 position of guanine are inferior substrates for hNudt16 [12]. In the case of IDP, a very rapid hydrolysis reaction was observed. However, two factors made it impossible to correctly determine the hydrolysis rate: the very rapid progress of the reaction exceeding the capacity of the HPLC equipment and the formation of the IMP product, which had an inhibitory effect on the reaction. In brief, the results obtained from hydrolysis showed that hNudt16 has a high specificity towards IDP and GppG. The other compounds were hydrolyzed at several times slower reaction rates.

### 2.2. Assessment of the Compliance of the Properties of hNudt16 Mutants with the Wild-Type Protein by Circular Dichroism (CD), Differential Scanning Fluorimetry (DSF)

The problem encountered in quantifying the rate of IDP hydrolysis and the inability to compare GppG with hydrolysis product of known binding affinity as GMP or IMP prompted us to seek other methods to assess the Nudt16 specificity for studied compounds. Insights into this process can be gained by measuring the interaction of the protein with the ligand. As hNudt16 is a hydrolyzing enzyme, its binding affinity to the compound can only be measured if the catalytic activity is impaired. Therefore, we searched for hNudt16 variants with reduced hydrolytic activity and retained substrate binding capacity. Previously, a single mutation in position E76Q and double mutations of glutamic acid at positions 79 and 80: EE-QQ and EE-KK have been described in the literature as hydrolytically inactive. These amino acids belong to the NUDIX motif and are involved in the coordination of Mg^2+^ ions in the structure. As a result, they have been proposed to play a role in the catalytic mechanism [2,17,25].

#### 2.2.1. Comparison of the Secondary Structure of hNudt16 and Its Mutants by CD

**Figure 2:**
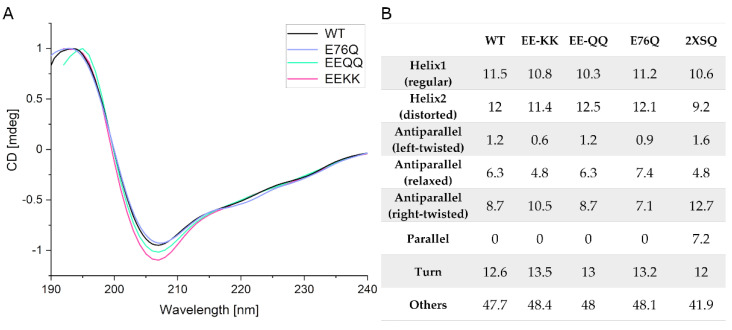
(**A**) CD spectra for wild-type hNudt16 and studied mutants. (**B**) Contribution of secondary structures in the polypeptide chain obtained from CD measurements (calculated in BeStSel) in comparison to the corresponding crystal structure.

To ensure that the chosen mutant is an appropriate substitute for the wild-type protein, we compared their physicochemical properties and structural features with those of the wild-type. Structural studies on the hNudt16 enzyme and E76Q, EE-QQ, and EE-KK mutants were performed using far-UV CD spectroscopy (Figure 2A). Analysis of the secondary structure content in wild-type hNudt16 in comparison to all tested mutants and the corresponding crystal structure of hNudt16 (2XSQ) was performed using BeStSel (Beta Structure Selection) web server [31,32]. Analysis of the CD spectra for the single (E76Q) and double (EE-KK and EE-QQ) hNudt16 mutants compared to the CD spectra for wild-type protein indicated that the overall secondary structural content for all mutants tested was not significantly different from that of the wild-type protein, and the CD spectra of hNudt16 and hNudt16 E76Q showed maximum visual overlap (Figure 2A). [31,32]. In all the tested samples, there were fewer β-type structures compared to the crystal structure (Figure 2B). Notably, for all hNudt16 proteins in solution parallel β-sheets were not detected by the BeStSel algorithm—that is, they no longer matched the structural parameters and were consequently classified by BeStSel as part of the “others” structures.

#### 2.2.2. Analysis of Protein Thermal Stability by DSF

The structure of hNudt16 reveals two Mg^2+^ ions, detailed analysis of their location in the structure suggests that a single mutation of E76Q may allow one of the magnesium ions to be retained, while a double mutation of EE-QQ and EE-KK is likely to affect the binding of both ions. Magnesium ions are essential for hydrolysis and are probably also important for substrate binding to the active site. We assumed that for further studies of the hNudt16-ligand interaction we require a mutant that would still contain magnesium ions, but only those that might be needed for substrate binding. We therefore tested the thermal stability of different mutants in the presence of magnesium ions, as their ability to bind these ions can be determined from T_m_ parameter.

We tested the effects of mutations introduced into the hNudt16 amino acid chain at positions 76, 79, and 80 on the thermal stability of the protein in the presence and absence of magnesium ions. Wild-type hNudt16 without magnesium ions had a melting point of T_m_ = 52.8 ± 0.1 °C. The introduced mutations resulted in a higher melting point for all mutants, with T_m_ of 56.0 ± 0.1 °C for E76Q, 62.5 ± 0.1 °C for EE-QQ, and 71.0 ± 0.1 °C for EE-KK. A thermal stability study of hNudt16 and its mutants in the presence of increasing magnesium concentrations (Figure S2) showed that the EE-KK double mutation at positions 79 and 80 prevented magnesium binding (ΔT_m_ = 0 °C at all magnesium concentrations used), whereas the EE-QQ mutation significantly reduced magnesium binding (ΔT_m_ < 3 °C). The negative effect on magnesium ions binding, which is probably essential for substrate binding of the wild-type protein, led us to exclude the EE-QQ and EE-KK mutants as candidates to replace the wild-type protein for further ligand binding studies.

On the other hand, in the E76Q mutant, increasing the magnesium concentration was found to gradually increase the melting temperature (ΔT_m_ > 10 °C at a minimum of 10 mM MgCl_2_) and magnesium ion saturation occurred at higher concentrations (20 mM MgCl_2_) than for wild-type hNudt16 (Figure S2). This indicates that the E76Q mutant is likely to bind the magnesium ion responsible for the catalysis process weaklier or not at all, while the binding of the other magnesium ion is retained and is therefore a suitable candidate for further protein–ligand studies. The hydrolytic properties of E76Q, EE-QQ, and EE-KK towards GppG and the E76Q mutant towards m^7^GppG were tested by high-performance liquid chromatography (HPLC), and none of the mutants exhibited hydrolytic activity (Figure S1).

### 2.3. hNudt16 Stability in the Presence of Different Ligands and Its Substrate Affinity Studies

#### 2.3.1. hNudt16-Ligand Complex Thermal Stability Screen by DSF and NanoDSF

Changes in protein behavior resulting from the formation of complexes with even weakly binding ligands usually affect the thermal stability of proteins. DSF is widely applied for early stage of drug discovery studies and obtained melting temperature (T_m_) is a parameter enabling hit molecules selection [33]. This technique allows high-throughput screening of molecules in easy and inexpensive way. An undoubted advantage, especially in case of dye-free nanoDSF, is the use of protein without the need for labeling or immobilization. To track changes in the behavior of hNudt16 in the presence of the tested ligands, the thermal stability of the hNudt16 E76Q mutant with various ligands was investigated using a DSF assay. Two different methods were used, namely DSF with SYPRO Orange dye and in parallel dye-free nanoDSF (using Prometheus NT.48), to exclude the potential influence of the SYPRO Orange dye on the resulting T_m_ values. Measurements were carried out under the same conditions in both the DSF and nanoDSF assays, with the same selected concentration for all tested ligands (0.5 mM). The results are shown in Table 2.

In the presence of SYPRO Orange, the average melting temperature (T_m_) of the apo protein was 1.3 °C lower than that of nanoDSF, indicating that the dye had little effect on the thermal stability of hNudt16 [34]. The ΔT_m_ values obtained by both methods were in agreement, and the order of the ligands in terms of ΔT_m_ increase were similar (Table 2, Figure S3). In both types of measurements, hNudt16 E76Q showed the highest increase in T_m_ in the presence of GppG and IDP; the high ΔT_m_ for these two ligands (approximately 17.5–18.0 °C relative to the apo form) (Table 2). For the ligand concentration used (0.5 mM), given the fact that ligand chemotype binds to the protein such a large shift in T_m_ indicates creation of the specific ligand:protein complex. A slightly weaker T_m_ shifts were observed for m^7^GppG and GDP, followed by GpppG, IMP, and m^7^GpppG, while the remaining compounds (GMP, dpCoA, NADH, AppA, CDP, m^7^GDP, NAD, and ADPr) only negligibly affect the thermal stability of protein of interest. The obtained data allowed us to restrict the list of compounds for further studies mainly to those reaching ΔT_m_ above 3 °C.

Furthermore, comparing to the DSF data for wild-type hNudt16 measured in the same conditions (in 20 mM MgCl_2_) using nanoDSF, the ΔT_m_ obtained in measurement with GMP was 3.5 ± 0.1 °C (T_m_ = 70.8 ± 0.1 °C) and with ADPr of 0.9 ± 0.1 °C (T_m_ = 68.1 ± 0.1 °C), which is very similar to the data obtained for the E76Q mutant. This allow us to conclude that there is no negative effect of the mutation on protein stabilization by studied compounds.

#### 2.3.2. Characterization of hNudt16-Ligand Complexes by Microscale Thermophoresis (MST)

**Figure 3:**
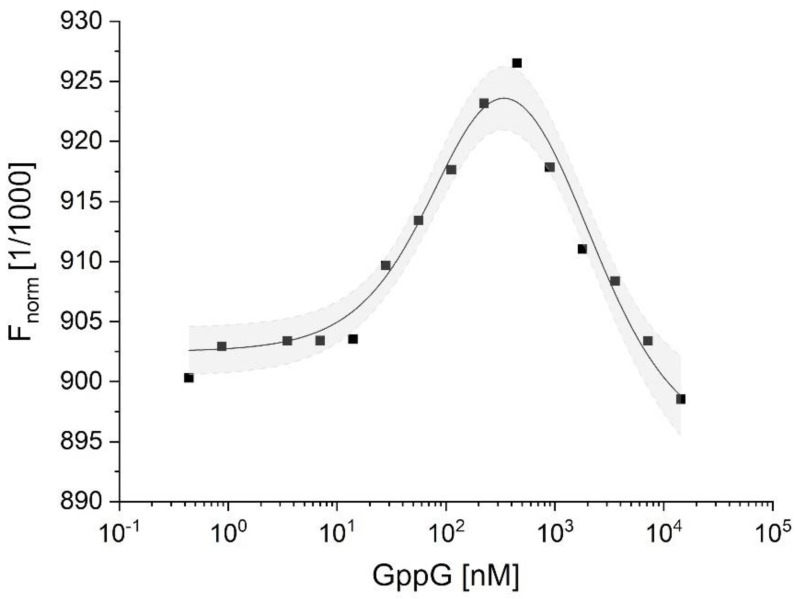
Representative MST pseudo-titration data for binding of GppG to hNudt16E76Q. Squares represent experimental points, solid lines represent results of fitting for two independent binding sites model, and gray area bounded by a dashed line represents 95% confidence bands for this model. See Figure S4 for other ligands data.

Next, we determined the direct binding affinities of a selected set of compounds that showed significant ΔT_m_ score when measured by the DSF assay using MST. Dissociation constants were estimated from a series of at least six pseudo-titration experiments for each ligand. The obtained MST curves showed two well-separated inflection points (Figure 3 and Figure S4). The first inflection point at lower ligand concentrations corresponds to a stronger binding and is most likely the major mode of protein–ligand binding. The second inflection point, at several orders of magnitude higher ligand concentrations, indicates the existence of a second, weaker binding site. Therefore, all data were analyzed using a global fit for a model with two independent binding sites [35].

**Figure 4:**
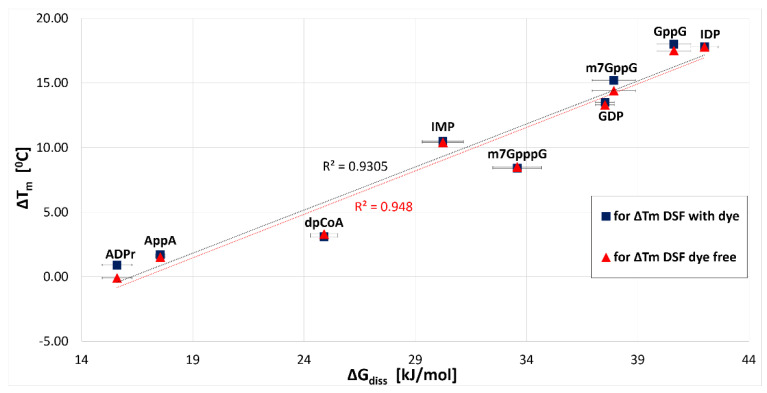
Comparison of the free energy associated with the dissociation of the complex (ΔG_diss_) calculated for stronger binding site (with K_d1_ obtained from MST) with ΔT_m_ obtained from dye-free and DSF with dye measurements for tested hNudt16E76Q-ligand pairs.

The K_d1_ and K_d2_ values obtained for all tested ligands are summarized in Table 3. Similar to previous experiments, the MST assay indicated that GppG and IDP were the best ligands with the highest affinity for hNudt16E76Q (K_d1_ values below 100 nM). The obtained dissociation constants are in agreement with the DSF data, as the free energy of dissociation (ΔG_diss_ calculated from measured K_d1_) correlate perfectly (R^2^ = 0.95) with ΔT_m_ obtained by DSF/nanoDSF measurements (Figure 4). Importantly, the stronger IMP binding constant (K_d1_ = 4.9 ± 1.8 µM for hNudt16E76Q) is in agreement with the K_d_ value (K_d_ = 5.24 ± 0.01 µM) obtained by Tresaugues using isothermal titration (ITC) for wild-type hNudt16 [17]. The consistency of these results supports our conclusion that the E76Q mutant retains ligand-binding properties analogous to the wild-type hNudt16 protein. Given the consistency of the MST results with DSF, we did not measure for some ligands that were very weakly bound or not bound by hNudt16E76Q. Obtained results allow us to conclude that hNudt16E76Q binds very strongly both compounds: IDP and GppG. While m^7^GpppG, IMP, dpCoA, AppA, ADPr exhibit weak affinity of several orders of magnitude.

#### 2.3.3. Determination of the Structure of hNudt16 in Solution by SAXS

**Figure 5:**
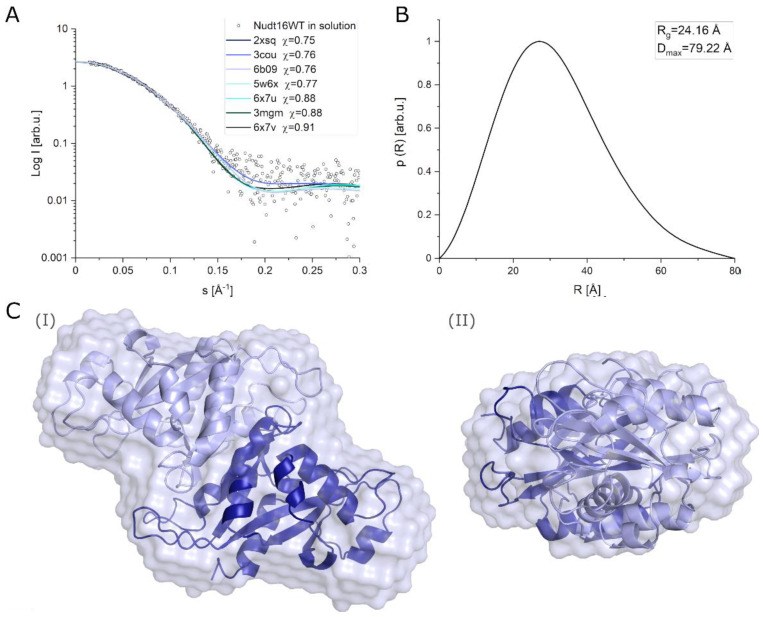
SAXS data analysis for hNudt16: (**A**) Comparison of experimental SAXS data for hNudt16 in solution with a fit for all crystal structures of hNudt16 dimer available in the PDB database. Theoretical scattering curves for PDB structures and discrepancy χ value (compared to SAXS data) were obtained in CRYSOL. PDB: 2XSQ had the lowest discrepancy with experimental SAXS data; (**B**) pair distance distribution function (*p*(R)) determined based on SAXS scattering curve for the hNudt16 (calculated in GNOM software). The calculated structural parameters were R_*g*_ = 24.16 Å and D_max_ = 79.22 Å; (**C**) superposition of low-resolution 3D model of hNudt16 in solution (semitransparent shape) with the crystal structure of dimeric hNudt16 PDB: 2XSQ in projections along the two axes of the molecule. Monomer-like subunits of 2XSQ dimer are marked with different colors (light and dark blue). Figures obtained in PyMOL.

To understand why we obtained two binding constants in MST studies we decided to examine the structure of the hNudt16. Several crystallographic structures of this protein are available, which can be used to analyze the molecular docking of the ligand of interest. The interaction studies were performed in solution therefore we decided to analyze whether the tertiary and quaternary structures of hNudt16 in solution were significantly different from those in the crystal structures. The SAXS data confirmed that hNudt16 in solution occurs in a stable dimeric form. Analysis of the SAXS data yielded the structural parameters of hNudt16 in solution. The radius of gyration (R*_g_*) calculated by fitting experimental data to the Guinier equation was R*_g_* = 24.16 ± 2.74 Å. The analysis of the pair distance distribution function *p*(R) in GNOM resulted in an identical value of R*_g_* = 24.17 ± 2.74 Å, and the maximum size of enzyme molecule D_max_ = 79.22 Å (Figure 5B).

First, we compared the experimental SAXS data obtained for hNudt16 in solution with all available crystal structures of hNudt16 (3COU, 6X7V, 3MGM, 6X7U, 5W6X, 6B09, and 2XSQ). Using CRYSOL software, we calculated the theoretical scattering curves for all crystal structures and compared them with the experimental curve (Figure 5A). This analysis showed that hNudt16 structures have a favorable fit to SAXS data with discrepancy in the range χ = 0.75 ÷ 0.91. The best fit with a discrepancy χ = 0.75 was obtained for the 2XSQ PDB. Structures with the lowest discrepancy from the experimental data in solution were most similar to the wild-type state of a protein. As a result, the 2XSQ was used to model the protein–ligand binding in the study of molecular docking.

Based on SAXS scattering curves, we performed ab initio modeling of the hNudt16 enzyme in solution using DAMMIN software [36]. The resulting low-resolution model of the hNudt16 dimer in solution is presented in Figure 5C. We performed calculations using DAMMIN with no constraints on the local symmetry of the dimer. Twenty independently received models were averaged using the DAMAVER program. Figure 5C(I,II) show a superposition of the obtained low-resolution 3D model of hNudt16 in solution (semitransparent shape) with the crystallographic 2XSQ in projections along the two different axes of the molecule. The crystal structure of hNud16 fits very well with the low-resolution bead model.

#### 2.3.4. Molecular Docking Analysis

**Figure 6:**
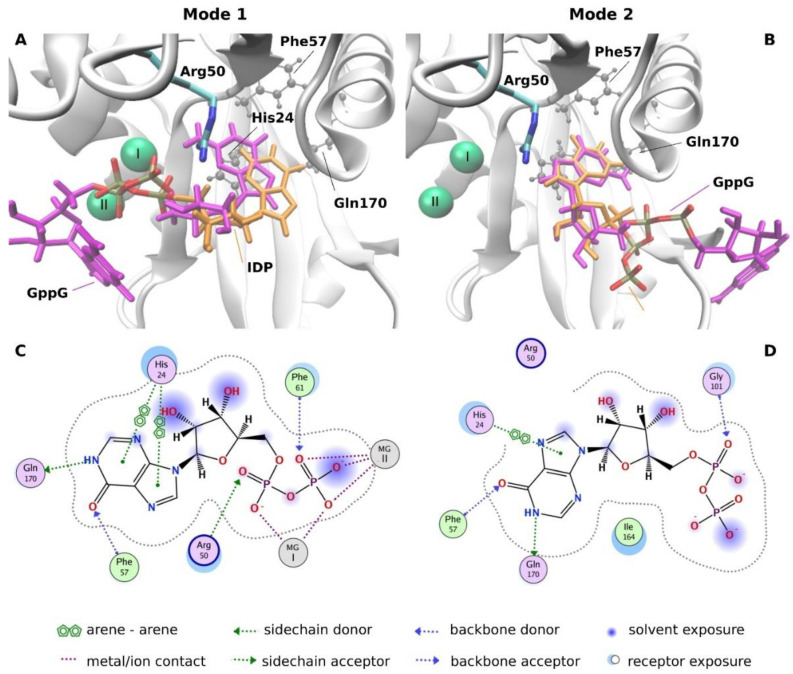
Top: IDP and GppG bound to hNudt16 in docking (**A**) mode 1 and (**B**) mode 2. Bottom: Schematic illustration of IDP interactions in (**C**) mode 1 and (**D**) mode 2.

To elucidate the structural reasons for the large differences observed in ligand affinities to hNudt16 dependent on the ligand nucleobase, we performed molecular docking of the chosen ligands to the hNudt16 crystal structure. The following three ligands were docked: IDP and GppG, which were found to strongly interact with hNudt16E76Q, and AppA, which showed weaker interactions with hNudt16E76Q (Table 2 and Table 3). Docking revealed two distinguishable ligand binding modes (Figure 6) inside the hNudt16 binding pocket, differing in the location of the phosphate groups and one of the nucleobases. All predicted ligand poses can be classified as one of these modes. In both modes, one of the bases was located inside the pocket flanked by His24, Phe57, and Gln170. The same pocket was occupied by the nucleobase of IMP, crystallized with hNudt16 (Figure S5). His24 interacts with the base via π stacking, while Phe57 and Gln170 participate in a hydrogen-bond network stabilizing the interacting ligand. The estimated free energies of binding in mode 1 of approximately −16 kcal/mol are two times more favorable than in mode 2 with ΔGs of −6 to −8 kcal/mol (Table S1).

**Figure 7:**
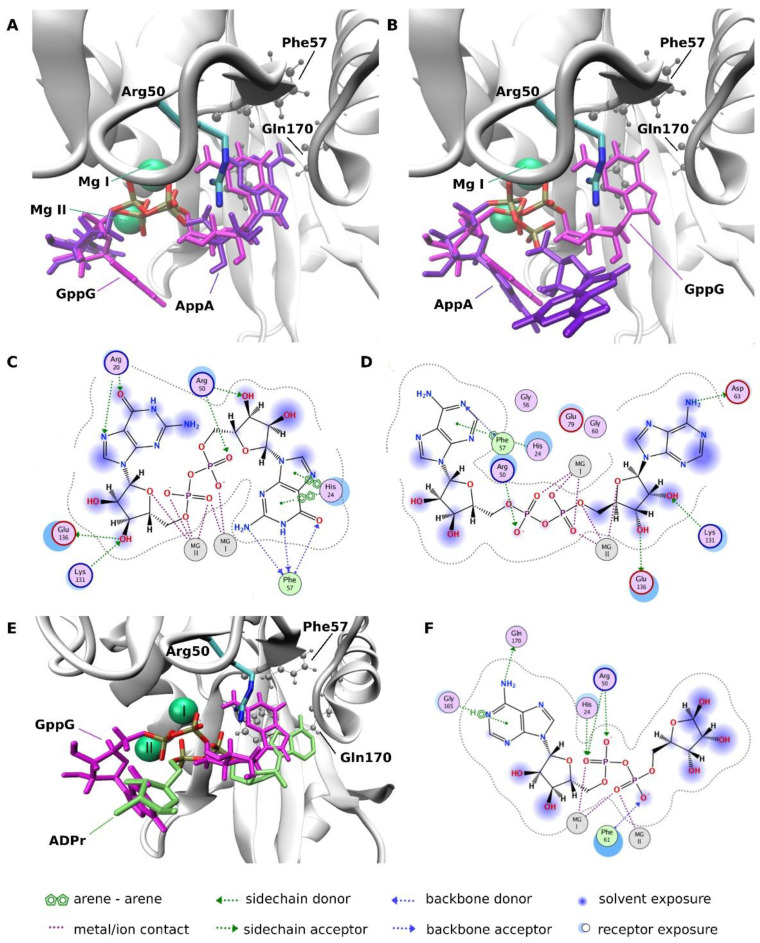
(**A**,**B**) Conformations of GppG and AppA bound to hNudt16 in mode 1. GppG remains in its active conformation, whereas adenine positions are less stabilized and one of the AppA adenines either stays inside (**A**) or flips out (**B**) of the pocket (marked with three residues, His24, Phe57 and Gln170 in ball and stick representation in gray). (**C**,**D**) Schematic illustration of GppG and AppA interactions in active conformations of mode 1 (ligand conformations as in (**A**)). (**E**) Comparison of the ligand positions in active conformations of mode 1: a very good hNudt16 substrate, GppG, vs. the conformation of a poor substrate, ADPr (ADPr position is from the PDB:5W6X structure). (**F**) Schematic illustration of ADPr interactions with hNudt16.

In mode 1, which was found to be energetically favored in the case of all docked ligands, two Mg^2+^ ions were closely aligned with the phosphate groups, showing strong charge-charge interactions (Figure 6 and Figure 7). Arg50, which appeared indispensable in the adoption of mode 1 conformations, in the most energetically favorable poses, bonded with the phosphate groups and ribose ring via hydrogen bonds (Figure 7). Therefore, we believe that Arg50 plays a vital role in positioning the ligands along the magnesium cations. In mode 1, the ligands interacted with structural elements that are crucial for the catalytic activity of hNudt16, such as Mg^2+^ and Arg50. In binding mode 1, this was likely the ligand conformation adopted during the cleavage event.

In mode 2, the phosphate groups and one of the bases moved away from the magnesium cations towards the exit of the hNudt16 active site. However, in mode 2, the ligand conformation and its interaction network varied and were not as conserved as those in mode 1. In dinucleotide ligands, one of the nucleobases fit into the hNudt16 pocket (Figure 6), while the other nucleobase adopted a range of conformations, interacting with various amino acids on the hNudt16 surface, including Gly101, Ser102, Ala4, Ser 166, and His99 (Figure S6).

All three ligands were found to dock in the two modes described above. However, docking showed that, in the case of AppA, even though AppA phosphates interacted with Mg^2+^ (as in binding mode 1), adenines were destabilized (Figure 7). While one of the adenines was matched to G in the GppG conformation (Figure 7A), in some cases, the other adenine was flipped outside of the binding pocket (Figure 7B). The charge distribution of AppA was different due to the fact that its base lacked the carbonyl group present in the well-interacting IDP and GppG ligands, which resulted in a weaker hydrogen bond network both inside the pocket and with the critical Arg50 residue (Figure 7D). Fewer interactions with key amino acids were also observed in the case of ADPr (ADPr position was taken from the PDB:5W6X structure) (Figure 7E,F). Although the interaction with Arg50 and His24 was still present, the hydrogen-bond network with these residues was weaker. His24 interaction with the base via π stacking and interaction with Phe57 was missing. The comparison of the ligand position in mode 1 in the case of very good hNudt16 substrates, such as IDP or GppG, with the conformation of poor substrates, such as AppA and ADPr, indicates that adopting nucleobase position inside the pocket flanked by His24, Phe57, and Gln170 and its stabilization by π stacking interaction may be crucial for strong binding and enzymatic catalysis.

## 3. Discussion

According to previous reports, hNudt16 can hydrolyze the canonical cap of cytoplasmic mRNAs and small nucleolar RNAs, eliminate dIDP/IDP and dITP/ITP, and process ADP-ribosylation of proteins, DNA, and RNA, as well as non-canonical metabolites present on some cellular RNAs [5,8,9,10,11,13,14,15,17,18,19,20,21,22,23]. However, it is difficult to determine whether all of the activities listed are biologically relevant. Particularly that some of the published results contradict each other [24]. To gain a better understanding of the activity of hNudt16, the present study focused on a quantitative comparison of the specific properties of hNudt16 with previously published substrates, as well as other substrates that have not yet been investigated for this protein. In our study, we screened and compared 15 compounds: GppG, GpppG, m^7^GppG, m^7^GpppG, GMP, GDP, m^7^GDP, IMP, IDP, CDP, dpCoA, AppA, ADPr, NAD, and NADH.

Both the wild-type protein and its mutants were investigated. Biophysical studies of different hNudt16 variants were performed to identify a variant that was able to bind the substrate but not hydrolyze, namely E76Q. The E76Q variant was the optimal replacement for the wild-type protein and was used as a model for a non-hydrolytically active protein. Our biophysical studies showed that the variant had an unchanged secondary structure and thermal stability. E76Q also showed the ability to bind magnesium ions, but most likely at only one position. The fact that the E76Q mutation did not affect ligand binding indicates that one magnesium ion coordinated by Glu80 [17] is sufficient for ligand binding. However, the mutation of any of the amino acids involved in the coordination of the second magnesium ion will impair the catalytic mechanism.

Protein–ligand binding studies with hNudt16E76Q showed that this mutant has the highest binding affinity towards IDP and slightly lower, but of the same order of magnitude, for GppG. Furthermore, wild-type hNudt16 had very high hydrolysis rate in activity screening assays with GppG. The DSF and MST results obtained for mutant E76Q with the tested ligands were consistent with results obtained from enzymatic assays for wild-type protein, and lower hydrolysis parameters were directly related to weaker binding affinity. Ligands with diphosphate bonds have a few orders of magnitude higher affinity than mono- and tri-phosphates for identical nucleotides, making the diphosphate substrates the most favored by hNudt16. Thus, we can conclude that the number of phosphate residues was essential for hNudt16 binding affinity and hydrolytic efficiency. This is consistent with the structure of X29 (homolog of hNudt16 from *X. laevis*) with GTP, where terminal phosphate (Pγ) is surrounded by acidic amino acids that may be involved in the formation of an acid patch that negatively affects the binding of triphosphate nucleotides in comparison to its diphosphate counterpart [17,25]. Interestingly, ligands with unmethylated nucleotides are not only hydrolyzed by hNudt16 with greater efficiency, as reported previously by Grzela et al. [12] but they also have higher binding affinity and thermal stability than methylated ligands. Binding affinity and hydrolytic studies showed that ligands such as NAD, NADH, free ADPr, or dpCoA are poor substrates for the hNudt16 enzyme (which was also shown in the case of radioactively labeled NAD [13], or ADPr with K_M_ ~ 1 mM [19]). hNudt16 has been reported to hydrolyze NAD and dpCoA moieties in vitro and in vivo when they are attached to 5′-RNA (metabolite caps) [13], or polyADPribosylated proteins [19]. This suggests that effective hydrolysis of these structures may depend on the interaction of hNudt16 with the remaining part of the processed molecule (e.g., RNA body). A potential positively charged channel for RNA binding was proposed based on the crystal structure of X29 [25]. However, we did not observe that the presence of the RNA chain had a significant effect on the rate of hydrolysis of the bound compound [12]. In contrast, GppG, which was shown here for the first time as a potent substrate for hNudt16, is an interesting compound for further research on the biological function of this protein because of its high affinity for hNudt16, similar to IDP. Dinucleoside diphosphates are compounds stored in dense granules in human platelets and released into the extracellular space. Jankowski et al. showed that GppG has a stimulatory effect on vascular smooth muscle cells, representing a new class of mediators with regulatory roles [27]. Dinucleoside polyphosphate are a part of the purinergic signaling system [28] similarly as guanidine-based purines (GTP, GDP, GMP, GUO, GUA). The latter have been shown to modulate important intracellular processes such as growth, differentiation and survival [29]. Given the hydrolytic activity of hNudt16, it may affect the level of compounds that are important components of the purinergic signaling system (e.g., GppG/GMP). Further research in this direction can bring important data about Nudt16 biological role.

The ligand-binding modes were elucidated to explain the structural reasons for the large differences in ligand-dependent affinities. Docking of the high-affinity ligands to hNudt16 indicated the existence of two ligand-binding modes. The presence of two binding modes is in agreement with the in silico identification of a potential secondary druggable site for hNudt16 by Michel et al. [37]. Both modes detected in this study are located inside the hNudt16 main binding pocket in the vicinity of the enzyme’s active site. Mode 1 is most likely an active conformation adopted during the catalytic event, as the ligands in mode 1 share a common position of phosphates in close proximity to the magnesium ions. They interact with structural elements that are crucial for the catalytic activity of hNudt16, such as Arg50 [17]. We also believe that Arg50 plays a vital role in positioning of the ligands along the magnesium cations, crucial for adopting an active conformation inside the hNudt16 catalytic pocket. The electrostatic attraction between the negatively charged phosphate groups and two Mg cations is a key constituent of the ligand binding pose in docking mode 1. A lack of both Mg^2+^ ions may prevent ligand binding in mode 1 and, as a result, inactivate enzyme.

Our experiments have shown two distinct binding affinity values for strongly interacting IDP and GppG. In line with experimental data, molecular docking identified two binding modes, with significantly different protein interaction energies. The element common to both modes is the binding of one of the bases in the pocket flanked by His24, Phe57 and Gln170. Because of the considerable overlap between these two modes, the enzyme can only bind one substrate at a time in the active site pocket. In this scenario, the two Kd values could mean that simultaneously a certain population of ligands is bound in mode 1 and another smaller population in mode 2. Another explanation could be a two-step ligand binding. In this case, some ligands would bind directly in mode 1, while others would initially bind in mode 2, in which one of the bases is stably bound in the enzyme pocket while other occupies a variety of positions outside the pocket. Such binding, being less stable, is likely to oscillate between different positions, to eventually flip to the stable and energetically favorable mode 1.

In the case of poorly bound and hydrolyzed ligand-AppA, we found that its interactions with hNudt16 in mode 1 are less stable; the nucleobase can easily flip out of the binding pocket, while still interacting with magnesium ions. This observation confirms that adopting a stable nucleobase position inside the pocket flanked by His24, Phe57, and Gln170 is crucial for enzymatic catalysis. The instability of AppA base, potentially resulting from less compatible charge distributions, is a probable cause of the weaker enzymatic processing of this ligand. Moreover, we found that Mg^2+^ ions are crucial for adopting an active conformation inside the hNudt16 catalytic pocket. The electrostatic attraction between the negatively charged phosphate groups and the two Mg cations is a key constituent of the ligand binding pose in docking mode 1. A lack of both Mg^2+^ ions may prevent ligand binding in mode 1 and, as a result, inactivate the enzyme.

Moreover, SAXS data confirmed that hNudt16 in solution occurs in a stable dimeric form. Based on SAXS scattering curves, we performed ab initio modeling and, as a result, we proposed a low-resolution 3D model of the dimeric hNudt16 structure in solution. The evaluation of the solution scattering for available crystal structures of hNudt16 (performed in CRYSOL based on PDB files) showed good fit for all crystal structures to SAXS data, wherein the best fit was obtained for the 2XSQ PDB structure. Thus, 2XSQ could be considered most similar to the native state of the protein.

## 4. Materials and Methods

### 4.1. Expression and Purification of hNudt16

Human Nudt16 (hNudt16) WT and its mutants (E76Q, EE-QQ, EE-KK) were expressed and purified using an adapted protocol from Wojtczak et al. [38]. The protein was expressed in *E. coli* Rosetta 2 (DE3) using the pET16b_Nudt16 vector. The construct contains four histidines, introduced just after the two C-terminal histidines of hNudt16, which resulted in a C-terminal 6xHis-tag. Starter LB culture with 34 μg/mL chloramphenicol and 100 μg/mL ampicillin was inoculated using a stock of *E. coli* Rosetta 2 (DE3) that had been transformed with the pET16b_Nudt16 vector and incubated at 37 °C overnight. Ten milliliters of starter culture was used to inoculate each 1 L of LB culture supplemented with 100 μg/mL ampicillin and 34 μg/mL chloramphenicol.

*E. coli* Rosetta 2 (DE3) cells were grown at 37 °C until the OD_600_ reached a value in the range of 1–1.5. Then, protein expression was induced by the addition of IPTG (0.2 mM), and the cells were incubated overnight at 18 °C with 180 rpm. Cells were harvested by centrifugation at 7700 rpm, washed in PBS, and collected again by centrifugation at 6000 rpm.

Next, the cell pellets were resuspended in a lysis buffer (20 mM HEPES-KOH pH 8.0, 50 mM NaCl, 300 mM urea, 10 mM imidazole, 10% glycerol, 1% Triton X-100, and 1 mM MgCl_2_) supplemented with lysozyme and 1000 U Benzonase or Viscolase, incubated on ice for 45 min, and disrupted by sonication. After centrifugation (25,000× *g* for 55 min), the supernatant was filtered and incubated at 4 °C overnight with gentle stirring in the presence of 5 mL HIS-Select Nickel Affinity Gel (Sigma-Aldrich, Saint Louis, MO, USA) equilibrated with binding buffer. Ni beads with bound hNudt16 were loaded into a gravity column (Bio-Rad, Hercules, CA, USA) and washed with 10-fold the column volume of buffer containing 20 mM Tris–HCl (pH 8.0) and 300 mM NaCl. His-tagged hNudt16 protein was eluted with a linear gradient of imidazole (20 mM to 300 mM) in wash buffer. Fractions containing pure hNudt16 (determined by SDS-PAGE) were pooled, dialyzed overnight at 4 °C in 20 mM HEPES pH 7.9, 300 mM NaCl, 10 % glycerol, and 0.5 mM TCEP, flash frozen in liquid nitrogen, and stored at −80 °C.

### 4.2. Site-Directed Mutagenesis of hNudt16

Human Nudt16 amino acid-substituted mutants (E76Q, double mutations of glutamic acid at positions 79 and 80; EE-QQ; and EE-KK) were obtained via site-directed mutagenesis using the QuikChange Site-Directed Mutagenesis Kit (Stratagene California, La Jolla, CA, USA). The DNA primers used for mutagenesis are listed in Table S2. The starting DNA template pBMH-Nudt16 plasmid was used (where the *hNudt16* coding sequence, with a sequence encoding four additional histidines introduced just after the two C-terminal histidines of hNudt16, was cloned between NcoI and BamHI restriction sites) [12]. Mutagenesis reactions were performed in a 50-μL final volume containing 1× Pfu polymerase reaction buffer (10 mM KCl, 10 mM (NH_4_)_2_SO_4_, 20 mM Tris-HCl (pH 8.8), 2 mM MgSO_4_, 0.1% Triton X-100, and 0.1 mg/mL BSA), 50 ng template plasmid DNA, 125 ng of forward and reverse primers, 1 μL of 10 mM dNTP, and 1 μL (2.5 U) of Pfu DNA polymerase. The PCR cycling conditions were as follows: 95 °C for 2 min, 12 cycles of 95 °C for 30 s, 55 °C for 1 min, and 68 °C for 7 min and 15 s, and a final incubation at 4 °C in the case of single mutation E76Q. In the case of double mutations (EE-QQ and EE-KK), the number of cycles was increased to 18 (instead of 12). Next, methylated plasmid DNA was digested for 1 h at 37 °C with 1 μL of DpnI restriction enzyme, and then 10 μL of DpnI-treated mix was transformed to chemicompetent *E. coli* XL1-Blue cells. Plasmid DNA was isolated from selected colonies obtained after transformation using QIAprep Spin Miniprep Kit (QIAGEN GmbH, Hilden, Germany) and analyzed by DNA sequencing for desired mutations. Finally, the *hNudt16* coding sequences with confirmed mutations were re-cloned into the pET16b expression vector as NcoI-BamHI restriction DNA fragments, and verified by DNA sequencing.

### 4.3. Chemical Synthesis of Cap Analogs

IMP, IDP, GMP, GDP, GTP, dpCoA, ADPr, NAD, and NADH were purchased from Sigma-Aldrich (Saint Louis, MO, USA). The synthesis of mono- (m^7^GDP) and dinucleotide cap analogs (m^7^GppG, m^7^GpppG, GppG, and AppA) was performed as previously described [39,40,41].

### 4.4. Circular Dichroism (CD)

Circular dichroism (CD) in the far-UV spectral region was performed using Chirascan Plus (Applied Photophysics Ltd., Leatherhead, UK) for the wild-type hNudt16 protein and the mutants E76Q, EE-QQ, and EE-KK. The spectra were acquired in a nitrogen atmosphere in a thermostatically regulated cuvette (set to 20 °C) with an optical path = 0.1 mm (High Precision Cell; Hellma Analytics GmbH & Co. KG, Müllheim, Germany) at the measurement parameters: χ in range 185 to 263 nm, with 0.5 nm step and 0.5 s time per step. Protein samples were added at a concentration of 0.74 mg/mL to buffer (10 mM Tris pH 7.9, 50 mM NaClO_4_, 0.2 mM TCEP, and 2 mM MgCl_2_). The buffer was filtered through a 0.22-µm filter, and both buffer and protein samples were degassed before measurement. Measurements were performed in six replicates for protein samples and 10 replications for buffer. The corresponding smoothed buffer spectrum was subtracted from the recorded spectrum of the sample solution. The buffer spectra were smoothed by the Savitzky–Golay method (window size 15) [42,43]. The data processing procedure was performed using machine included software Pro-Data Chirascan 4.1 (Applied Photophysics Ltd Leatherhead, United Kingdom). Analysis of the secondary structure content was performed using the BeStSel server [31].

### 4.5. Small Angle X-ray Scattering (SAXS)

The SAXS patterns for hNudt16 in solution were recorded using the XEUSS 2.0 SAXS/WAXS laboratory beamline (XENOCS, Grenoble, France) equipped with a MetalJet microfocus X-ray source (λ = 0.134 nm) with a liquid metal-jet anode (gallium alloy) (Excillum AB, Kista, Sweden) and a Pilatus 3R 1M hybrid photon counting detector (Dectris, Baden-Daettwil, Switzerland). Briefly, 50 µL of a hNudt16 solution at a concentration of 2.618 mg/mL in buffer (20 mM HEPES pH 7.9, 300 mM NaCl, 10% glycerol, 0.5 mM TCEP, and 20 mM MgCl_2_) was injected manually into the low-noise liquid flow cell. The experimental scattering vector range was 0.0125 Å^−1^ < *s* < 0.58 Å^−1^ (*s* = (4π/λ) sinϴ, where ϴ is the scattering angle). The measurements were conducted at room temperature, and the scattering data were collected over 120 min as a series of 12 frames (600 s per frame). SAXS data reduction and processing were performed using Foxtrot 3.2.7 [44] and the PRIMUS program from the ATSAS package [45]. The radius of gyration (R_*g*_) was calculated using PRIMUS software. The molecular weight of the hNudt16 samples was obtained from the extrapolated I(0) value compared to that of bovine serum albumin as a standard [46]. The pair distance distribution function (*p*(R)) and maximum particle dimension (D_max_) were calculated using GNOM [47,48]. A comparison of the obtained experimental scattering data for hNudt16 with the theoretical scattering curves calculated for hNudt16 crystal structures available in PDB database was performed using CRYSOL and data truncated to 0.3 Å^−1^ [49] The low-resolution dummy atom 3D model of hNudt16 in solution was obtained with the ab initio modeling method using DAMMIN and data truncated to 0.3 Å^−1^ [36].

### 4.6. Differential Scanning Fluorimetry (DSF)

#### 4.6.1. Protein Thermal Stability Screen

The protein thermal stability of wild-type hNudt16 and all tested mutants in the presence of increasing Mg^2+^ ion concentration was measured using DSF with SYPRO orange dye on CFX RT-PCR (Bio-Rad, Hercules, CA, USA). This method is based on the change in fluorescence of the SYPRO orange dye in an aqueous buffer, which is strongly quenched, while its binding to the exposed hydrophobic regions of thermally unfolded protein results in an increased fluorescence [50]. The optimal concentrations of protein, SYPRO orange dye, and buffer composition, in particular ion content, were determined. The samples were prepared and measured in clear 96-well multiplate PCR plates for RT-PCR (Bio-Rad, Hercules, CA, USA). The final conditions in the measured wells were 5 µM protein, 4× SYPRO orange dye, and an increasing MgCl_2_ concentration, all in a buffer consisting of 10 mM HEPES pH 7.9, 150 mM NaCl, 5% glycerol, and 1 mM TCEP. The individual components of the solution were added in equal volumes to each well, with a final sample volume of 25 μL/well. The samples were gently mixed, and the 96-well plate was covered with optically clear PCR sealing tape (Bio-Rad, Hercules, CA, USA) to prevent evaporation. Samples were equilibrated at 25 °C for 5 min, and then the thermal unfolding of the protein was monitored using a temperature ramp of 1 °C/min in the range of 25–95 °C (fluorescent reads were taken every 30 s). The data processing procedure was performed using Bio-Rad CFX Manager (version 1.6.541.1028), and melting point temperatures were calculated as the minimum of the first derivative plot.

#### 4.6.2. hNudt16-Ligand Thermal Stability Screen

Protein thermal stability in the presence of various ligands was measured using two DSF methods: assays with SYPRO orange dye and dye-free assays. DSF assays with SYPRO orange dye were performed at specific ligand concentrations for each protein–ligand pair. This measurement was performed as described in Section 4.6.1, with a constant MgCl_2_ concentration of 20 mM and a concentration of the tested ligand maintained at 0.5 mM. DSF dye-free measurements were performed using a Prometheus NT.48 nanoDSF device (NanoTemper Technologies, München, Germany). The composition of the samples was identical to that in the DSF with SYPRO orange dye measurements, without the addition of any dye. The conditions during measurements were as follows: 5 µM protein, 20 mM MgCl_2_, 10 mM HEPES pH 7.9, 150 mM NaCl, 5% glycerol, 1 mM TCEP, and 0.5 mM ligand. The measurements were performed at a single ligand concentration for all ligands. After 10 min of pre-incubation, the samples were loaded into standard capillaries (NanoTemper Technologies, München, Germany). During the measurement, the samples were heated in a temperature range of 20–95 °C at a rate of 1 °C/min, and the measurements were performed with an excitation power of 30%. All of the collected data measured with using the above two DSF methods were analyzed according to the two-state transition numerical model described previously [51], using the Marquardt algorithm implemented in the Origin 2019 package (OriginLab, Northampton, MA, USA). We fitted the melting temperatures (T_m_) globally for at least three independent experiments (but separately for different DSF methods).

### 4.7. Microscale Thermophoresis (MST)

Binding constants for protein–ligand pairs were established using the MST method. The hNudt16 E76Q was labeled with RED-tris-NTA 2nd Generation dye (Monolith HIS-tag labeling Kit, NanoTemper Technologies, München, Germany) according to the manufacturer’s instructions. Measurements were carried out in a wide range of ligand concentrations, selected after preliminary tests for each of the ligands. The results obtained were used to determine of binding parameters for a given ligand. Each MST measurement consisted of a series of 16 serial 1:2 dilutions of the ligand, and was performed at a constant dye-labeled protein concentration of 50 nM. After 10 min of pre-incubation, dye-labeled protein–ligand samples were manually loaded into Monolith™ NT.115 Premium Capillaries (NanoTemper Technologies, München, Germany) and analyzed using the Monolith NT.115 (NanoTemper Technologies). As we observed two separated inflection points indicating two non-equivalent binding sites, analysis with standard NanoTemper software that enables using only two models, namely (1) assuming one binding site (or equivalent binding sites) and (2) cooperative binding sites (Hill Equation), was insufficient. Therefore, the results were analyzed using the model of two independent binding sites, as described previously by Winiewska et al. [35] Analysis was performed using Origin package (OriginLab, Northampton, MA, USA) and dissociation constants were fitted globally for a series of at least six MST pseudo-titration experiments for each ligand. Up to 10 replicates were performed for some ligands.

For the dissociation constants obtained, we also calculated the free energy associated with the dissociation of the complex ΔG_diss_ according to the equation: ${\Delta G}_{\mathrm{diss}}=-{\mathrm{RTlnK}}_{d}$ [52].

### 4.8. Enzymatic Assays

Enzymatic hydrolysis catalyzed by hNudt16 was performed at 37 °C in 40 mM Tris buffer (pH 7.9) containing 100 mM NaCl, 6 mM MgCl_2_, and 2 mM DTT. The concentration of the investigated dinucleotides and mononucleotides was 20 µM in the reaction mixture. The enzyme concentration depended on the type of substrate (0.002 µM for GppG, 0.008 µM for m^7^GppG, 0.08 µM for AppA, NADH, and dpCoA, 0.5 µM for m^7^GDP, or ADPr and NAD).

The hydrolytic susceptibility of the compounds was studied using HPLC. Before each experiment, 1 mL of buffer solution containing the analyte was incubated at 37 °C for 10 min. The hydrolysis process was initiated by the addition of hNudt16. To analyze the reaction progress, 200-µL aliquots of the reaction mixture were withdrawn and incubated at 96 °C for 5 min to stop the reaction through heat inactivation of the enzyme. The samples were then subjected to an HPLC system (Agilent 1200 series, Agilent Technologies, California, USA) with a reverse-phase Supelcosil LC-18-T column and UV/VIS detector. The substrates and hydrolysis products were eluted at 20 °C with a linear gradient of methanol in 0.1 M KH_2_PO_4_ (from 0% to 40%) over 15 min at a flow rate of 1.0 mL/min. Changes in the absorbance at 260 nm were continuously monitored during the analysis. Hydrolysis products were identified by comparing their retention times with those of the reference samples. The extent of decapping, determined as the percentage of the hydrolyzed substrate, was calculated using the area under the chromatographic peak of the respective compounds.

### 4.9. Molecular Docking

The hNudt16 crystal structure in complex with IMP and magnesium ions (PDB ID:2XSQ [17]) was used as a template for docking. The ligands were docked to hNudt16 using MOE software (version 2018) (Chemical Computing Group ULC, Montreal, QC, Canada). The hydrogen atoms were assigned using the MOE Protonate 3D function. The ligand models (IDP, GppG, and AppA) were manually prepared using the MOE molecule editing plugin. Dockings were conducted using a rigid receptor protocol. With the Triangle Matcher placement method, 100 ligand poses were identified, which were then refined and scored based on the binding free energies estimated using the GBVI/WSA dG algorithm [53]. For each ligand, docking experiments were conducted at least twice, each generating five ligand poses. VMD [54] and MOE Ligand Interaction plugins were used to generate the figures. The docking protocol was verified on the structure of the hNudt16-IMP complex (PDB ID: 2XSQ [17]) showing that the root-mean-square deviation (RMSD) between the conformation of the docked and crystallographic IMP (heavy atoms) was only 0.22 Å (Figure S5).

## Figures and Tables

**Table 1:** Hydrolysis of dinucleotides catalyzed by hNudt16; nd—hydrolysis products not detected at conditions used for dinucleotides. Data are presented as v/µg (hydrolyzed substrate (µM) per minute) per enzyme (µg) and as v/[E]_0_ (hydrolyzed substrate (µM) per minute) per [enzyme] (µM).

Dinucleotide	Hydrolysis Products	Hydrolysis Rate	
		(µM/min/µg)	(µM/min/[E]_0_)
GppG	GMP	227.27 ± 18.25	5454.5 ± 436.3
m^7^GppG	m^7^GMP + GMP	30.11 ± 2.81	722.6 ± 68.6
AppA	AMP	1.70 ± 0.12	40.8 ± 2.9
dpCoA	AMP + dpCop	0.75 ± 0.05	18.0 ± 1.3
NADH	AMP + NMNH	0.78 ± 0.06	18.7 ± 1.5
NAD	NMN + AMP	0.14 ± 0.02	3.4 ± 0.4
ADPr	nd	nd	
m^7^GDP	nd	nd	

**Table 2:** Comparison of thermal stability of hNudt16E76Q in the presence of various ligands, measured using two DSF methods. The following melting point values were obtained in conditions: 5 µM hNudt16E76Q, 20 mM MgCl_2_, 10 mM HEPES pH 7.9, 150 mM NaCl, 5% glycerol, 1 mM TCEP, and 0.5 mM ligand in dye-free DSF, as well as 4 × SYPRO Orange dye in the case of the DSF with dye method. Ligands with an increase of at least 3 °C in the T_m_ of hNudt16EQ are in bold.

	nanoDSF (Dye-Free)		DSF (with SYPRO Orange)	
Ligand	T_m_ (°C)	∆T_m_ (°C)	T_m_ (°C)	∆T_m_ (°C)
apo	65.1 ± 0.1	-	63.8 ± 0.1	-
**GppG**	83.1 ± 0.1	18.0 ± 0.1	81.2 ± 0.1	17.5 ± 0.1
**IDP**	82.9 ± 0.1	17.8 ± 0.1	81.5 ± 0.1	17.8 ± 0.1
**m^7^GppG**	80.2 ± 0.1	15.2 ± 0.1	78.2 ± 0.1	14.4 ± 0.1
**GDP**	78.6 ± 0.1	13.5 ± 0.1	77.0 ± 0.1	13.3 ± 0.1
**GpppG**	76.0 ± 0.1	10.9 ± 0.1	74.8 ± 0.1	11.0 ± 0.1
**IMP**	75.6 ± 0.1	10.5 ± 0.1	74.1 ± 0.1	10.4 ± 0.1
**m^7^GpppG**	73.5 ± 0.1	8.4 ± 0.1	72.3 ± 0.1	8.5 ± 0.1
**GMP**	69.1 ± 0.1	4.0 ± 0.1	68.0 ± 0.1	4.3 ± 0.1
**dpCoA**	68.1 ± 0.1	3.1 ± 0.1	67.0 ± 0.1	3.3 ± 0.1
NADH	67.4 ± 0.1	2.4 ± 0.1	64.9 ± 0.1	1.1 ± 0.1
AppA	66.8 ± 0.1	1.7 ± 0.1	65.2 ± 0.1	1.5 ± 0.1
CDP	66.8 ± 0.1	1.66 ± 0.1	65.6 ± 0.1	1.81 ± 0.1
m^7^GDP	66.7 ± 0.1	1.6 ± 0.1	64.4 ± 0.1	0.7 ± 0.1
NAD	66.1 ± 0.1	1.1 ± 0.1	63.7 ± 0.1	0.0 ± 0.1
ADPr	66.0 ± 0.1	0.9 ± 0.1	63.6 ± 0.1	−0.1 ± 0.1

**Table 3:** K_d1_ and K_d2_ binding constants obtained from MST and the free energy associated with dissociation of the complex (ΔG_diss_) calculated for each binding site (from K_d_ obtained by MST) for tested hNudt16 E76Q-ligand complex samples; nd—not determined.

Ligand	K_d1_ (nM)	K_d2_ (µM)	ΔG_diss1_ (kJ/mol) (for K_d1_)	ΔG_diss2_ (kJ/mol) (for K_d2_)
IDP	42 ± 11	1.2 ± 0.2	42.00	33.80
GppG	73 ± 23	1.6 ± 0.4	40.62	33.01
m^7^GppG	220 ± 87	30.2 ± 2.5	37.92	25.75
GDP	258 ± 45	45.6 ± 2.4	37.53	24.73
m^7^GpppG	(1.7 ± 0.6) × 10^3^	230 ± 57	33.58	19.32
IMP	(4.9 ± 1.8) × 10^3^	41 ± 10	30.24	24.98
dpCoA	(43 ± 11) × 10^3^	≥15,019	24.90	≤10.38
AppA	(833 ± 164) × 10^3^	nd	17.54	nd
ADPr	(1826 ± 497) × 10^3^	≥19,900	15.60	≤9.69

## Data Availability

The data presented in this study are available on request from the corresponding author.

## Supplementary Materials

All data are available online at https://www.mdpi.com/article/10.3390/ijms222010929/s1.

## Abbreviations

NUDIX	Nucleoside diphosphate linked to another moiety X
GDP	Guanosine diphosphate
GMP	Guanosine monophosphate
m^7^GDP	7-methylguanosine diphosphate
m_3_^2,2,7^GDP	2,2,7-trimethylguanosine
NAD+	Nicotinamide adenine dinucleotide
NADH	Reduced NAD+
FAD	Flavin adenine dinucleotide
dpCoA	Dephospho-coenzyme A
IDP	Inosine diphosphate
IMP	Inosine monophosphate
ITP	Inosine triphosphate
CDP	Cytidine diphosphate
ADPr	Adenosine diphosphate ribose
CD	Circular dichroism
SAXS	Small-angle X-ray scattering
DSF	Differential Scanning Fluorimetry
nanoDSF	Low-volume Differential Scanning Fluorimetry
TCEP	(Tris(2-carboxyethyl)phosphine)
MST	Microscale Thermophoresis
HPLC	High Performance Liquid Chromatography
PDB	Protein Data Bank
dNTP	Deoxynucleotide triphosphate
