# Supplementary material for: Insight into the Binding and Hydrolytic Preferences of hNudt16 Based on Nucleotide Diphosphate Substrates
Source: Int J Mol Sci. 2021 Oct 10;22(20):10929. doi: 10.3390/ijms222010929 (PMC8535469; doi:10.3390/ijms222010929)
Supplement: Supplementary file 1 [file ijms-22-10929-s001.zip › ijms-1376334-supplementary.pdf]

Supporting information

Insight into the binding and hydrolytic preferences of hNudt16 based on nucleotide containing diphosphate substrates

Magdalena Chrabąszczewska [1], Maria Winiewska-Szajewska [2], Natalia Ostrowska [3], Elżbieta Bojarska [3], Janusz Stępiński [3], Łukasz Mancewicz [1], Maciej Łukaszewicz [1], Joanna Trylska [3], Michał Taube [4], Maciej Kozak [4,5], Edward Darżynkiewicz [1,3], and Renata Grzela [1,3,*]

1. Division of Biophysics, Institute of Experimental Physics, Faculty of Physics, University of Warsaw, Pasteura 5, 02-093 Warsaw, Poland; Magdalena.Chrabaszczewska@fuw.edu.pl (M.C.); lu.mancewicz@gmail.com (L.M.); Maciej.Lukaszewicz@fuw.edu.pl (M.L.)
2. Institute of Biochemistry and Biophysics, Polish Academy of Sciences, Pawinskiego 5a, 02-106 Warsaw, Poland; mwin@ibb.waw.pl
3. Centre of New Technologies, University of Warsaw, Banacha 2c, 02-097 Warsaw, Poland; n.ostrowska@cent.uw.edu.pl (N.O.); e.bojarska@cent.uw.edu.pl (E.B.); j.stepinski@cent.uw.edu.pl (J.S.); joanna@cent.uw.edu.pl (J.T.); edward.darzynkiewicz@cent.uw.edu.pl (E.D.)
4. Department of Macromolecular Physics, Faculty of Physics, Adam Mickiewicz University, Uniwersytetu Poznanskiego 2, 61-614 Poznan, Poland; mtaube@amu.edu.pl (M.T.); mkozak@amu.edu.pl (M.K.)
5. National Synchrotron Radiation Centre SOLARIS, Jagiellonian University, Czerwone Maki 98, 30-392 Krakow, Poland

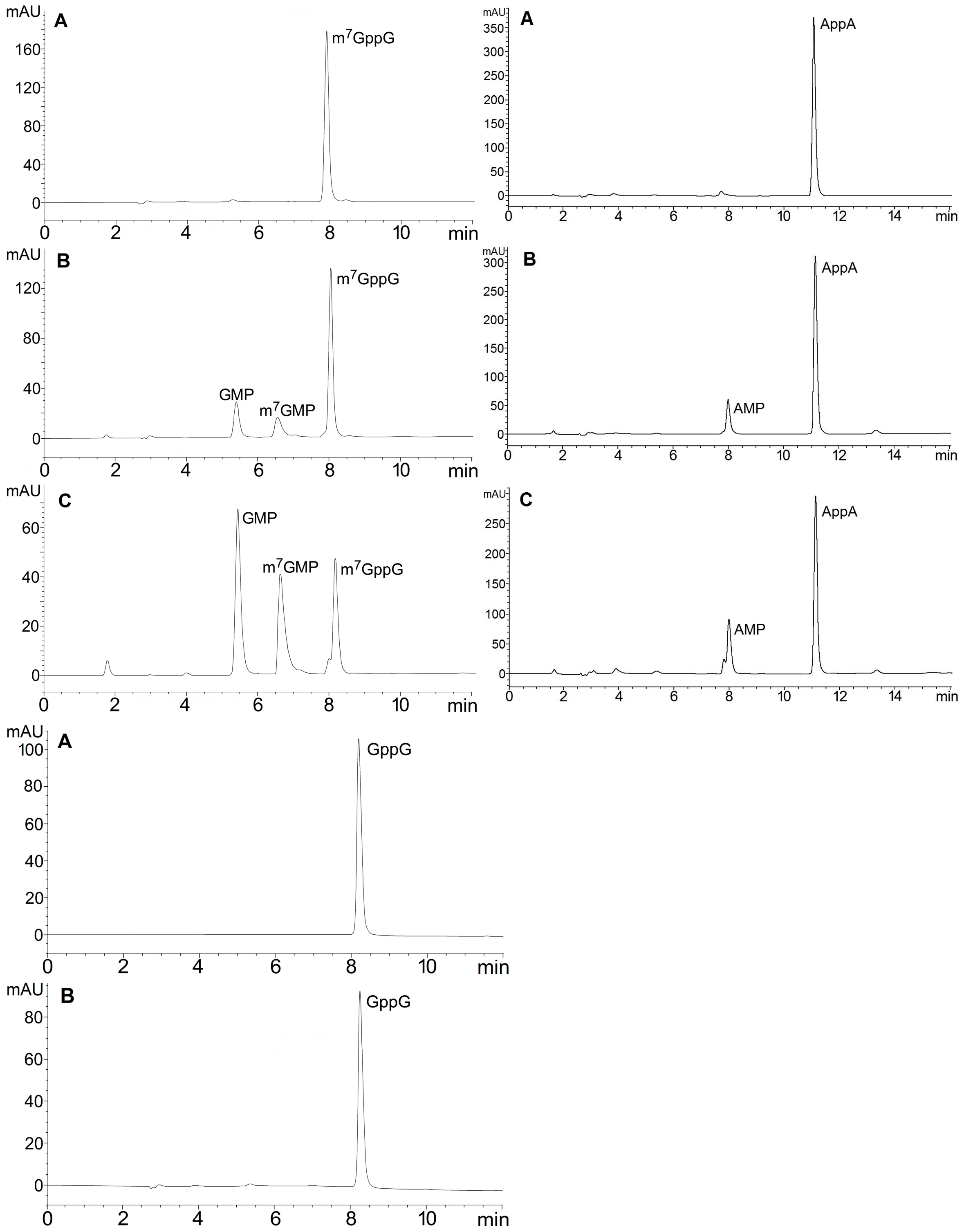

Figure S1. HPLC chromatograms of m^7GppG, AppA and GppG. Upper left panel: (A) m^7GppG in buffer (without hNudt16), (B) after 2 min reaction with hNudt16, (C) after 12 min reaction with hNudt16. m^7GppG concentration was 20 μM, enzyme concentration 0.008 μM. Upper right panel: (A) AppA in buffer (without hNudt16), (B) after 2 min reaction with hNudt16, (C) after 12 min reaction with hNudt16. AppA concentration was 20 μM, enzyme concentration 0.08 μM. Lower panel: (A) GppG in buffer (without hNudt16 E76Q), (B) GppG incubated with a mutant hNudt16 E76Q during 5 min.

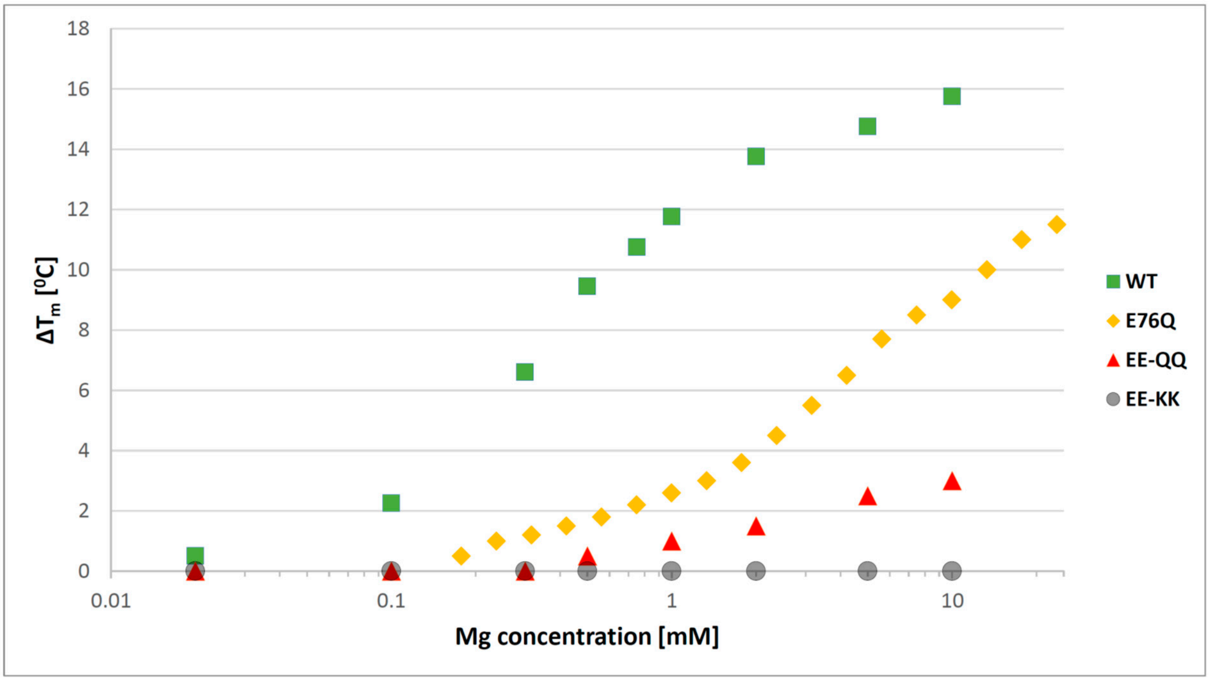

Figure S2 Melting point of hNudt16WT and tested mutants depending on the concentration of magnesium ions

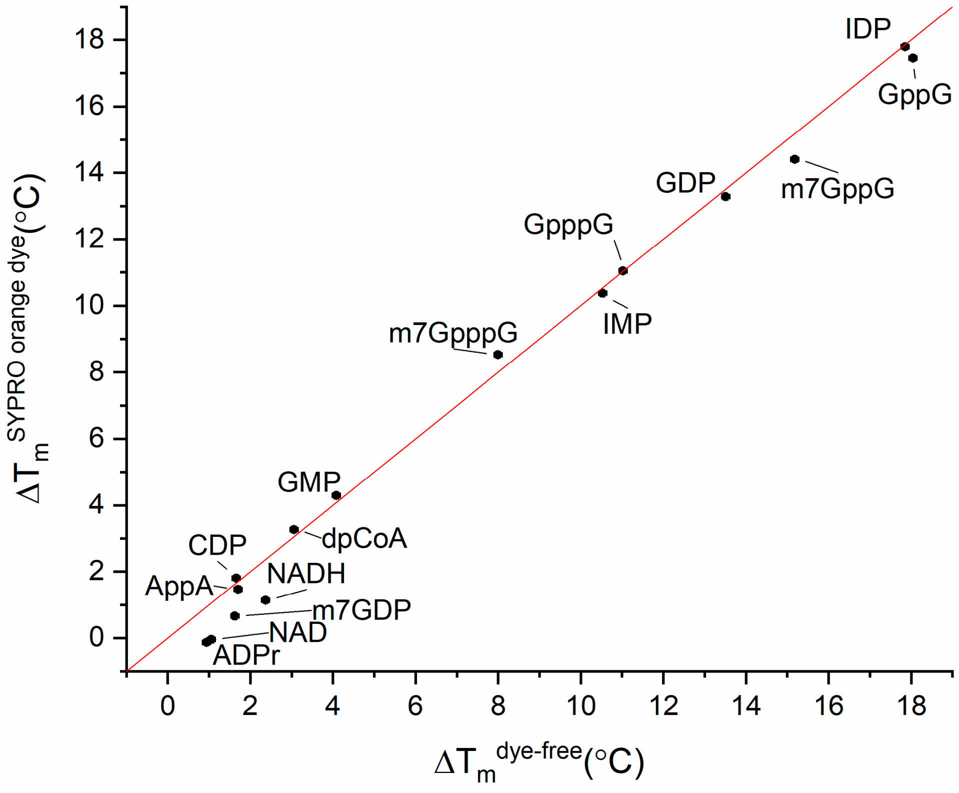

Figure S3. hNudt16 E76Q thermal stability in the presence of various ligands measured using two DSF methods: assay with SYPRO orange dye and dye-free (red line – 1:1)

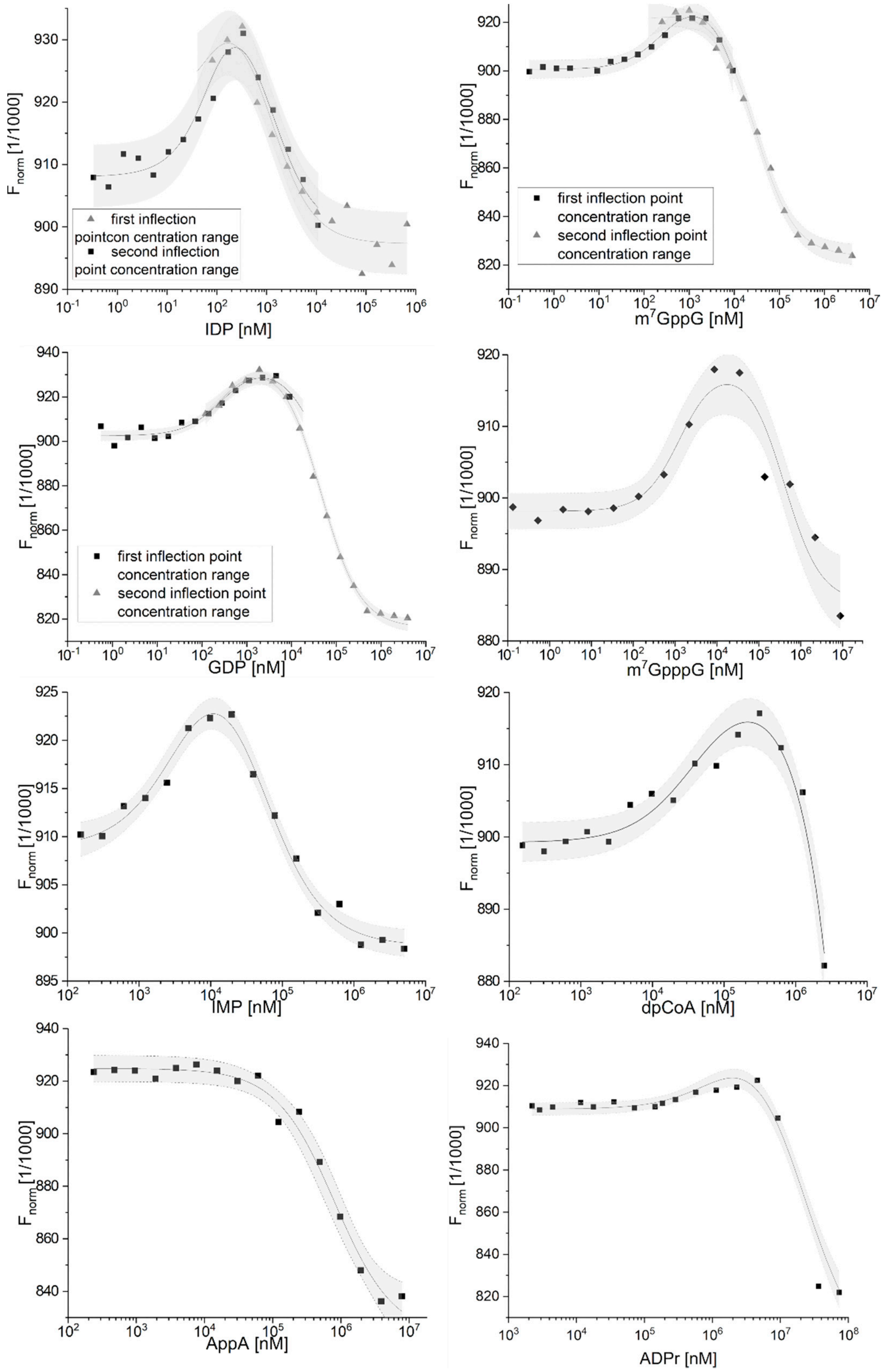

Figure S4. Representative MST pseudo-titration data for each ligand binding to hNudt16E76Q. Squares represent experimental points, solid lines represent results of fitting for two independent binding sites model, and gray area bounded by a dashed line represents 95% confidence bands for this model.

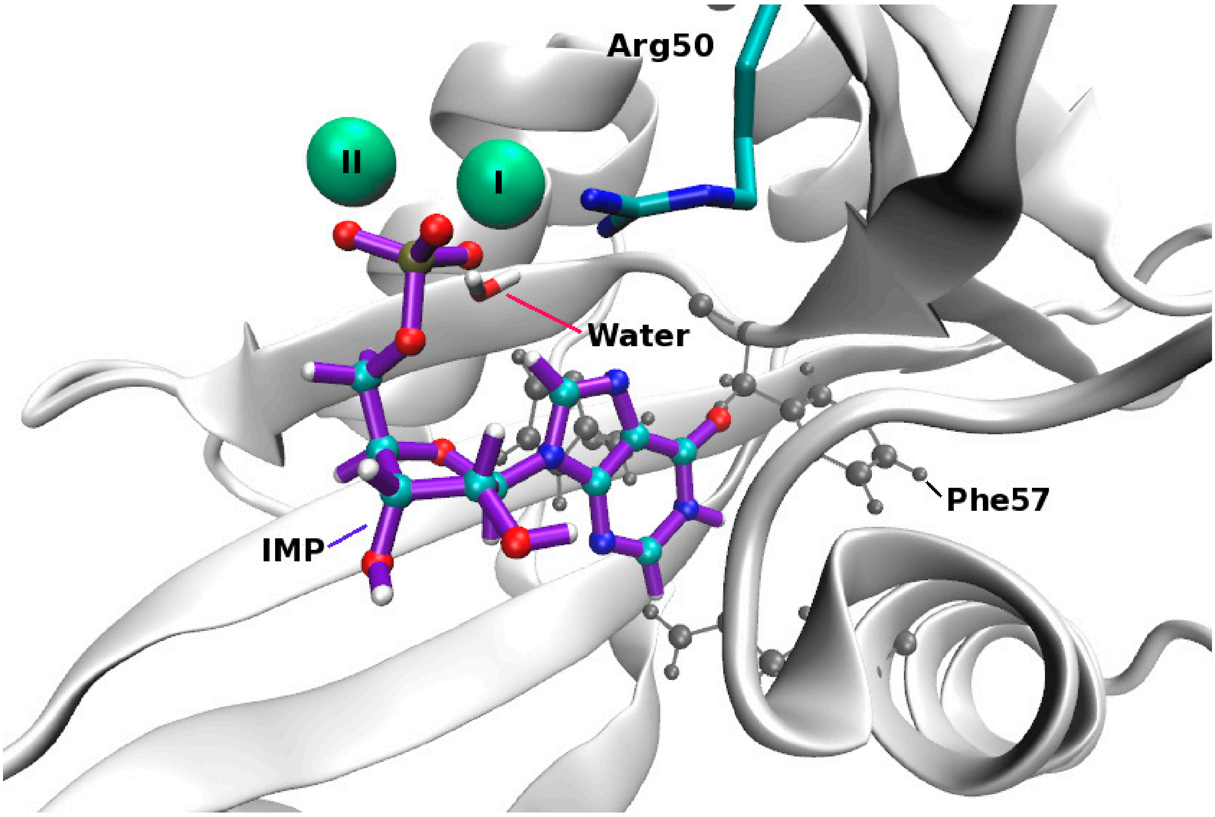

Figure S5. IMP crystallized with hNudt16 (PDB ID:2XSQ), with the ligand colored by atom types, compared with the position of IMP docked with MOE (in violet). Mg ions are shown in green and labelled I and II. IMP was docked to the enzyme with one crystallographically resolved water molecule (shown in the figure) that was found indispensable for this particular IMP-enzyme interaction. The heavy atom derived RMSD between the crystallised and docked ligand equals 0.2 Å meaning that the ligand conformations are nearly identical.

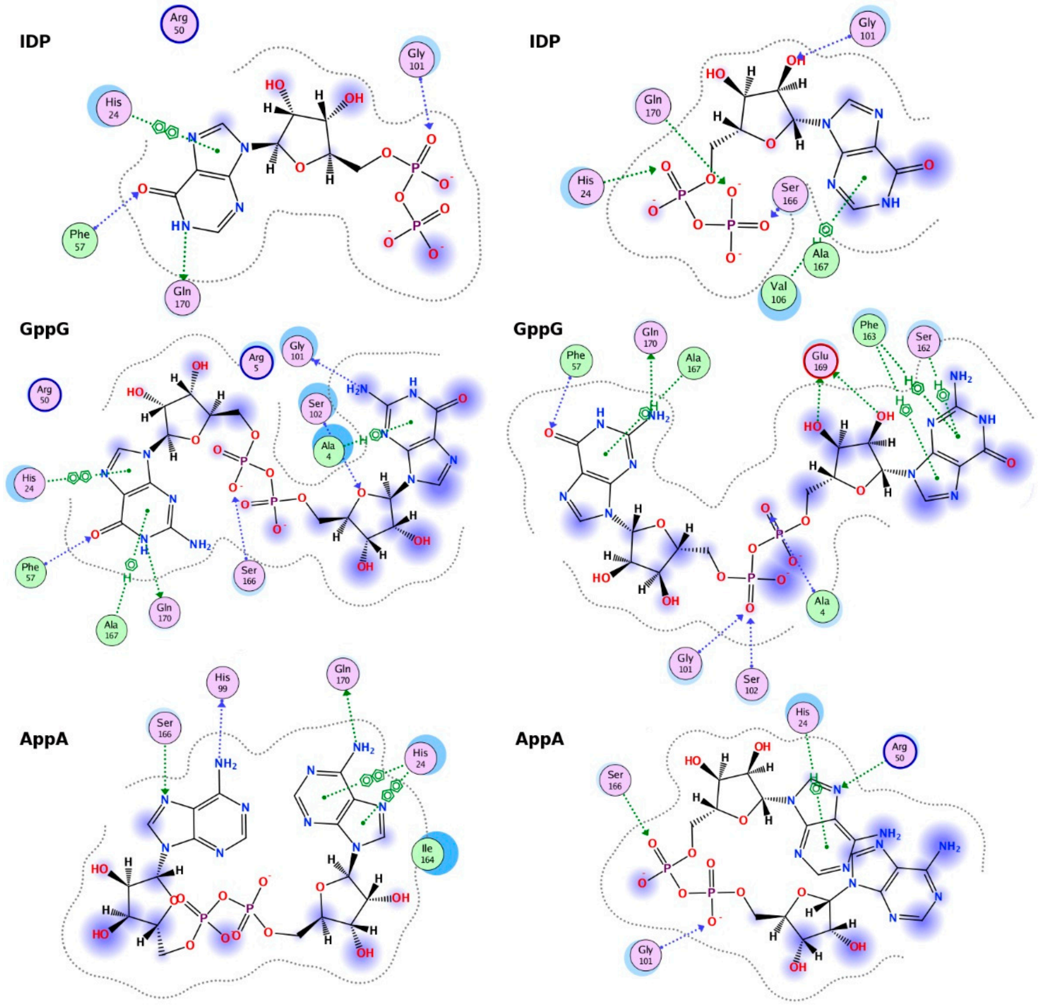

Figure S6. Possible interactions between hNudt16 and various ligands in binding mode 2 (not involving ligand contacts with magnesium ions). For each ligand, two representative conformations are shown. The conformations differ by nucleotide position, yet regarding the localization of one of the bases inside the pocket flanked by His24, Phe57 and Gln170, all conformations were classified into mode 2. In case of AppA, in the conformation on the right, the base is partially flipped outside hNudt16 binding pocket, and shifted towards Arg50 residue, while the conformations of IDP and GppG inside the pocket remain stable.

Table S1. MOE docking scores for the five docking poses in each mode. The AppA positions in which the phosphate groups interact with magnesium cations are marked with +. The position of AppA in mode 1 shown in Figure 2 in the main text is additionally marked with * where one of the nucleobases is flipped out.

	ΔG [kcal/mol]	
Ligand	Mode 1	Mode 2
IDP	-17.4	-7.5
	-17.1	-6.9
	-16.9	-6.6
	-16.9	-6.5
	-16.8	-6.4
GppG	-17.5	-8.9
	-17.0	-8.3
	-16.3	-7.9
	-16.2	-7.9
	-15.6	-7.7
AppA	-16.4 (+)	-8.5
	-15.0 (+) *	-8.1
	-14.9 (+)	-7.9
	-13.7	-7.8
	-12.5	-7.7

Table S2. Designed DNA primers used in mutagenesis

**Introduced mutation**	**Designed primers for site-directed mutagenesis**
Nudt16 E76Q (GAG→ CAG)	Forward: 5' C GGG CTG AAC CGC CAG CTG CGC GAG GAG 3'<br>Reverse: 5' CTC CTC GCG CAG CTG GCG GTT CAG CCC G 3'
Nudt16 EE79,80KK (GAG GAG → AAG AAG)	Forward: 5' CTG AAC CGC GAG CTG CGC AAG AAG CTG GGC GAA GCG GCT G 3'<br>Reverse: 5' C AGC CGC TTC GCC CAG CTT CTT GCG CAG CTC GCG GTT CAG 3'
Nudt16 EE79,80QQ (GAG GAG → CAG CAG)	Forward: 5' CTG AAC CGC GAG CTG CGC CAG CAG CTG GGC GAA GCG GCT G 3'<br>Reverse: 5' C AGC CGC TTC GCC CAG CTG CTG GCG CAG CTC GCG GTT CAG 3'
